# Non–English Language Resources and Readability of Kidney Transplant Center Websites in the United States

**DOI:** 10.1001/jamanetworkopen.2021.34236

**Published:** 2021-11-11

**Authors:** Robert Olmeda Barrientos, Valeria S. M. Valbuena, Clare E. Jacobson, Keli S. Santos-Parker, Maia S. Anderson, Seth A. Waits, Jessica R. Santos-Parker

**Affiliations:** 1 University of California Riverside School of Medicine; 2 Department of Surgery, Section of Transplantation, Michigan Medicine, University of Michigan, Ann Arbor; 3 University of Michigan Medical School Ann Arbor

## Abstract

This study examined US kidney transplant center websites for readability and for inclusion of languages other than English.

## Introduction

English proficiency impacts a patient’s understanding and use of health information and correlates with health disparities and worse outcomes among kidney transplant patients.^1^ Online resources convey complex patient education but may be ineffective for non-English speakers or those with low reading levels. Kidney transplant center websites have not been assessed for non–English language content or readability. We evaluate non–English language resources and readability of kidney transplant center websites in the United States.

## Methods

We conducted a cross-sectional study systematically reviewing kidney transplant center websites in the United States identified through the Organ Procurement and Transplant Network (OPTN) in July 2020. Websites were evaluated for transplant-specific content of any non-English language including the home page, subpages, drop-down menus, and external links. Written-English content was extracted and analyzed for readability utilizing the Readability Studio Professional Edition program (2020, Oleander Software) for 4 validated formulas—Flesch-Kincaid Grade Level, Fry Readability Graph, Gunning Fog Index, and Simple Measure of Gobbledygook (SMOG). Wilcoxon signed rank tests compared readability levels across regions. Pairwise correlations of formula scores were performed with Bonferroni correction. Statistical significance was set at α = .05. Analysis was performed with R version 3.3.2. This nonhuman research study is exempt from institutional review board approval and follows Strengthening the Reporting of Observational Studies in Epidemiology (STROBE) reporting guidelines for cross-sectional studies.

## Results

**Figure 1.**
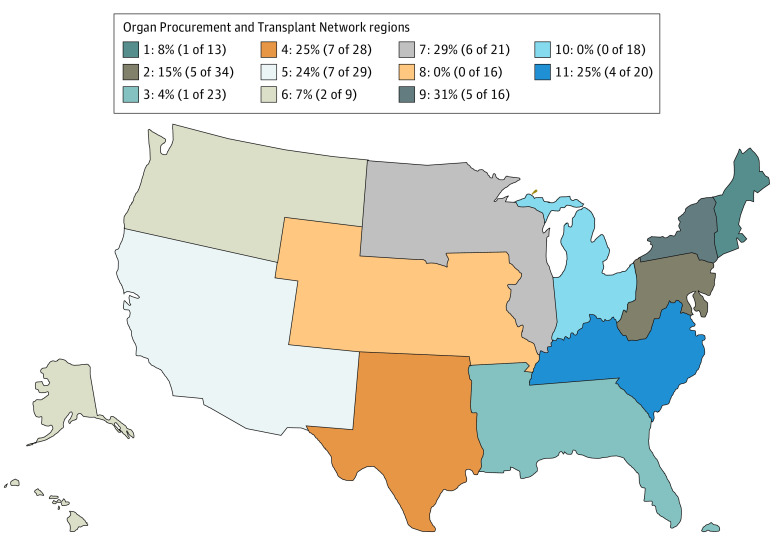
Proportion of US Kidney Transplant Center Websites With Alternative Language Resources

**Figure 2.**
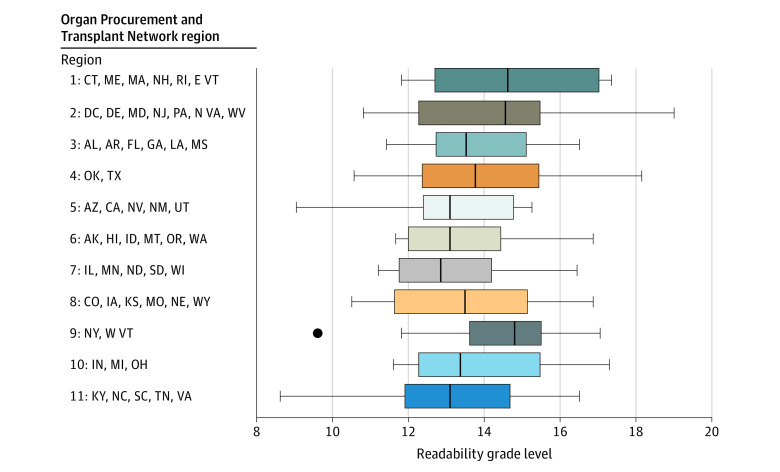
US Kidney Transplant Center Median Readability Scores The recommended reading level for kidney transplant informational material is in the sixth- to seventh-grade range.^2^ Dark lines represent the median; bar edges, 25% to 75% range; error bars, maximum and minimum values; and the black dot, outlier for having IQRs of more than 1.5. Not all regions follow state borders.

Of the 227 kidney transplant center websites, 38 (17%) provided resources in any non-English language (Figure 1). Two OPTN regions had no centers with additional languages: region 8, which had 16 websites, and region 10, which had 18 websites. With 31% (5 of 16), region 9 had the highest percentage of websites with resources in more than one language. For readability scoring, websites had to have a minimum amount of written content for the software to analyze (5 did not), and an additional 6 did not meet the Fry Readability Graph formula inclusion parameters, leaving 216 eligible for analysis. The median reading level of all websites was 13.6 (range, 8.6-19), which is the equivalent of ranging from an eighth- or ninth-grade reading level to a doctoral degree reading level, with no significant variation in median readability between regions (Figure 2). All readability tests were positively correlated (*P* < .001).

## Discussion

Just 17% of kidney transplant center websites provided English-language alternatives. Websites consistently scored at a college-reading level, well above the eighth-grade average reading comprehension in the United States. Sixth- or seventh-grade reading levels are recommended.^2^ These findings suggest that kidney transplant center websites are largely inaccessible, which may perpetuate health illiteracy and disparities in kidney transplants.

Health disparities in kidney transplants have remained prevalent in the United States despite ongoing efforts to overcome systemic racism.^3,4^ A recent study analyzing the readability of the top 10 websites from an online search for deceased and live kidney donation found that the content was written at a college level.^5^ Patient and family members’ understanding of transplant health information is critical for the self-advocacy and informed consent needed to reach the waiting list, understand information on posttransplant care, and know how to become a living donor. The few transplant centers that have been intentional in providing equitable information to Hispanic populations have found success through the creation of bilingual and culturally targeted websites about living-donor donation and transplant.^6^ Our findings suggest that greater intentional efforts along similar lines are needed nationally.

Readability scores are difficult to interpret and are highly variable, a main limitation of this study. However, the formulas used herein have been established for assessing written patient-facing content and have proved highly concordant across evaluated websites.^2^ Of note this study focused solely on transplant center websites, yet other general websites exist providing transplant information.

Current online resources must be adapted, new resources created with readability in mind, and materials translated into multiple languages to improve accessibility of online patient information. A centralized resource for accessible transplant education, made available in multiple languages and at an appropriate reading level could be linked to kidney transplant center websites nationally as a standardized first step.
